# Protein Aggregation Profile of the Bacterial Cytosol

**DOI:** 10.1371/journal.pone.0009383

**Published:** 2010-02-25

**Authors:** Natalia S. de Groot, Salvador Ventura

**Affiliations:** Departament de Bioquímica i Biologia Molecular, Universitat Autònoma de Barcelona, Barcelona, Spain; Swiss Federal Institute of Technology Lausanne, Switzerland

## Abstract

**Background:**

Protein misfolding is usually deleterious for the cell, either as a consequence of the loss of protein function or the buildup of insoluble and toxic aggregates. The aggregation behavior of a given polypeptide is strongly influenced by the intrinsic properties encoded in its sequence. This has allowed the development of effective computational methods to predict protein aggregation propensity.

**Methodology/Principal Findings:**

Here, we use the AGGRESCAN algorithm to approximate the aggregation profile of an experimental cytosolic *Escherichia coli* proteome. The analysis indicates that the aggregation propensity of bacterial proteins is associated with their length, conformation, location, function, and abundance. The data are consistent with the predictions of other algorithms on different theoretical proteomes.

**Conclusions/Significance:**

Overall, the study suggests that the avoidance of protein aggregation in functional environments acts as a strong evolutionary constraint on polypeptide sequences in both prokaryotic and eukaryotic organisms.

## Introduction

In the cellular context, it is the native protein fold that determines the biological function. Therefore, protein misfolding is usually associated with the impairment of essential cellular processes. In many cases, the assembly of misfolded polypeptides into cytotoxic aggregates mediates this deleterious effect. Accordingly, protein deposition is linked to the onset of more than 40 different human disorders [1]. In these diseases, proteins usually self-assemble into highly ordered, β-sheet enriched, supramolecular structures known as amyloid fibrils. However, the aggregation into amyloid conformations is not restricted to disease-related proteins but appears to be a generic property of polypeptides [2], [3], [4]. Moreover, although traditionally thought to be restricted to eukaryotic cells, recent studies provide compelling evidence for the formation of toxic amyloid assemblies inside bacteria [5], [6], [7], [8]. In this scenario, because all organisms face the important challenges of protein misfolding and aggregation, the existence of evolutionarily conserved strategies to avoid the deleterious effects of undesired protein deposition is likely.

The main intrinsic properties that determine protein aggregation have been defined and different computational approximations [9], [10], [11], [12], [13], [14], [15], [16], [17], [18], [19], [20] have exploited them to predict with reasonable accuracy the regions of proteins with the highest aggregation propensity, also called hot spots, as well as the overall protein aggregation propensity. Most of these algorithms only require the protein primary sequence as the input, allowing their easy implementation for the large-scale analysis of protein sets [1], [21], [22], [23], [24], [25], [26], [27]. Rosseau and co-workers used the TANGO algorithm to analyse the aggregation propensity of 28 complete proteomes, finding that polypeptides without a defined structure, and therefore with a solvent-accessible sequence, are less aggregation-prone than globular proteins [27]. The same group demonstrated that in *Escherichia coli* (*E. coli*), there is a bias towards the presence of residues with a low aggregation propensity flanking aggregation-prone stretches and that chaperones seem to have evolved to recognise these sequence features [27]. Tartaglia and co-workers employed their algorithm to compare the deposition tendency of different eukaryotic proteomes. They observed that the proteins of higher eukaryotes, and specifically of those with a longer lifespan, tend to be less aggregation-prone [24]. Moreover, the study of the *Saccharomyces cerevisiae* proteome revealed that in this organism, the protein aggregation propensity is associated to both protein function and localisation [23]. More recently, Chiti and co-workers used the Zyggregator program to analyse the aggregation tendency of the human proteome, their results recapitulated those of the above-discussed studies and additionally showed that long human proteins posses less-intense aggregation peaks than shorter ones [21].

Here, we have used AGGRESCAN, an algorithm previously developed by our group [10], [28], to analyse the aggregation propensity of the experimentally determined cytosolic proteome of the *E. coli* strain MC4100. This protein set comprises more than 1000 different proteins for which the individual abundance in the cytoplasmic fraction could be experimentally measured [29]. The results of our analyses provide new insights into the relationship between the intrinsic deposition propensities, cellular protein concentrations and protein expression regulation. In addition, the data recapitulate most of the previous observations on virtual proteomes. The overall analysis suggests that natural selection modulates proteins aggregation propensities according to their cellular function, structure, concentration and localization.

## Results and Discussion

Increasing evidence suggests that, in addition to protein function, protein solubility acts as a strong evolutionary constrain, so that any protein can remain functional in its native state under physiological conditions at its specific cellular localisation [30]. Many of the data supporting this view come from the analysis of the aggregation properties of theoretical proteomes derived from the predicted ORFs in different genomes. Bacterial organisms have long provided the bedrock on which to understand the complexity of protein folding and aggregation *in vivo* [31]. In the present work, we address the determinants underlying the aggregation properties of the real set of proteins that are present in the bacterial cytosol during exponential growth. Because these polypeptides coexist in time and space and their specific activities and relative abundance levels are the real effectors of cell function under such conditions, one might expect, in principle, that the evolutionary constrains modulating protein aggregation would become more evident in this specific protein group that when analyzing virtual proteomes, or even experimental transcriptomes, both of which do not necessarily represent the final complement of functional proteins present in a cell under particular, physiologically relevant, conditions. In addition, because the bacterial cytosol is the major cell factory for recombinant protein production, the information about the factors modulating protein aggregation in this specific compartment could be of biotechnological interest.

### AGGRESCAN Parameters and the Protein Data Set

**Figure 1:**
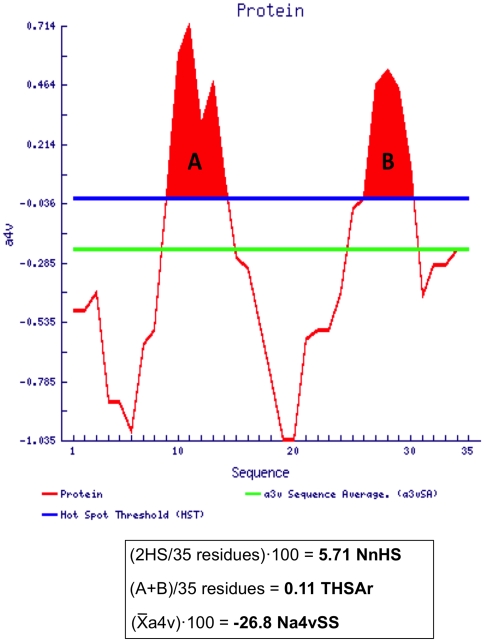
Example of AGGRESCAN output. The red line represents the aggregation profile of a putative protein with 35 amino acids. The blue line indicates the hot spot threshold, according to the individual aggregation propensity of the 20 natural amino acids and their frequency in natural proteins [28]. The green line corresponds to the average aggregation propensity of the putative protein. The aggregation-prone areas over the threshold are filled in red (A and B). a4v is the aggregation propensity average over a sliding window of 5 to 11 residues [10]. The aggregation propensity of each amino acid results from the depositional analysis of a set of amyloid polypeptides in the *E. coli* cytoplasm [25], [28].

AGGRESCAN is based in the use of a scale of amino acid aggregation propensities derived from experimental intracellular aggregation assays in living cells in the presence of the intact protein quality control machinery [25], [32], [33]. Because, *E. coli* was used as a model system to derive such scale, one might expect that the algorithm would provide accurate predictions for the aggregation properties of natural bacterial proteins expressed in the same cellular context, as those analyzed in the present work. From the different outputs provided by the program, in the present work we have selected the following parameters: the number of hot spots in a sequence (NnHS), the total area of these aggregation-prone regions (THSAr) and the global protein aggregation propensity (Na4vSS). We choose this particular set of values because, in AGGRESCAN, all of them are normalized relative to the number of amino acids in the sequence, allowing the direct comparison of proteins with different sizes (Figure 1).

The protein data set includes 1103 different proteins whose presence could be experimentally detected in the purified bacterial cytosol [29]. We curated the data by eliminating proteins that PSORT [34], [35] classified as belonging to other subcellular compartments (190) and those for which experimental evidences indicated that they were not or not mainly cytosolic (49). Similarly, proteins assigned by PSORT to other compartments but experimentally shown to be cytosolic (11), were included in the analysis, resulting in an 875 cytosolic polypeptide set. It is worth to mention, that 334 proteins in this set were classified by PSORT as having an unknown location. Because they have been experimentally identified in the cytosol we considered them to belong to this cellular compartment. Importantly, removing them from the cytosolic group does not change the results we obtained for this set (data not shown) and, accordingly, the complete 875 polypeptide set was used for all of the subsequent analyses, except for the calculation of the aggregation propensities of bacterial compartments, where the whole data set was employed. AGGRESCAN was run and the above-mentioned values were calculated for each protein in the set.

### The Cytosolic Proteins Abundance Correlate with Their Aggregation Propensity

Most protein aggregation processes follow a nucleation-polymerization scheme, in which the formation of the initial aggregation nuclei represents the rate-limiting step of the overall process. Nucleation processes correspond to second-order reactions and therefore the rate of protein aggregation is strongly dependent on the initial protein concentration. Therefore, the effective intracellular concentration becomes an important parameter when studying protein aggregation *in vivo*. The number of mRNAs in the bacterial cytosol encoding a given protein can vary from 1 to 100,000 [36]. Ishihama and co-workers developed the exponentially modified Protein Abundance Index (emPAI) to approximate the real concentration of a protein in a living cell. This index associates the number of mass spectrometry-sequenced peptides for each experimentally detected protein with its concentration in a given preparation. Later on, they applied this approach to successfully calculate the abundance of individual proteins in the bacterial cytosolic fraction [29], [37].

The aggregation properties of proteins appear to be associated to the specific cellular compartment where they reside [30], which makes sense because all the polypeptides in a given location feel the same environmental conditions. This suggests that the dynamic range of aggregation propensities in a given compartment cannot be very large. Therefore, to analyze if there is any relationship between the abundance and the aggregation of cytosolic proteins, we compared the aggregation features of the 10% most abundant proteins with those of the 10% least abundant ones according to their experimental emPAI values.

**Figure 2:**
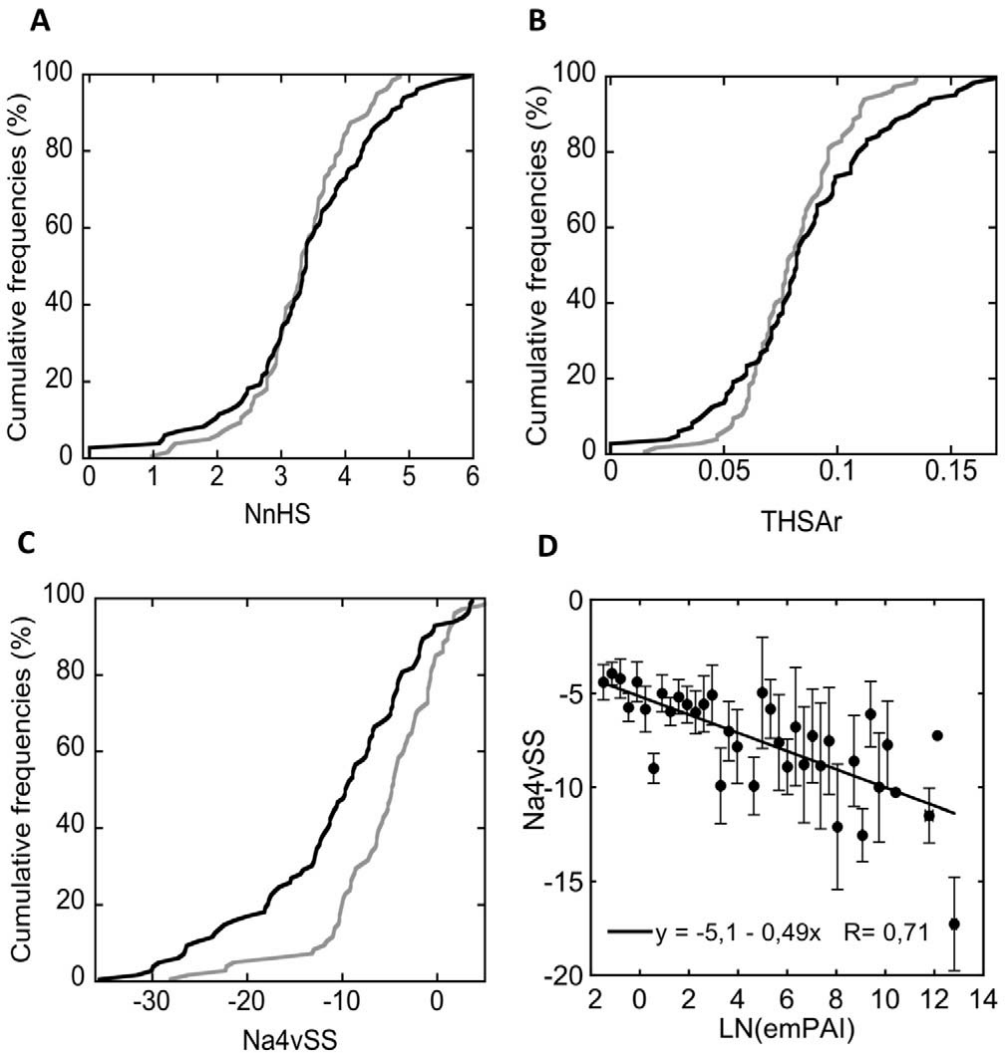
Relationship between the cytosolic proteins abundance and the AGGRESCAN aggregation parameters. Cumulative distributions of the NnHS (A), THSAr (B) and Na4vSS (C) parameters in the 10% most abundant cytosolic proteins (black) and the 10% least abundant ones (grey). D) Correlation between protein abundance, measured as LN(emPAI), and protein aggregation propensity, measured as Na4vSS, in the complete cytosolic protein set. The 875 cytosolic proteins were divided in 45 groups according to their LN(emPAI) values. Each point in the graphic represents the average value of the corresponding group. Standard errors for aggregation and abundance measurements are shown.

The normalized average number of aggregation-prone regions (NnHS) is approximately three in both groups. However, sequences devoid of any hot spot were observed only in the high-abundant group and sequences with NnHS values ≤2 were also more frequent in this subset (Figure 2A). Nevertheless, the frequency of proteins with NnHS values ≥5 was also higher in this group. The graphic of the THSAr closely resembles that of the NnHS, indicating that no important differences exist in the area associated with the aggregation-prone regions between the two groups (Figure 2B). In contrast, the overall aggregation propensity of low-abundant sequences is clearly much higher than that of high-abundant (Figure 2C).

To study the degree of association between the abundance of cytosolic proteins and their overall aggregation propensity, the complete 875 cytosolic protein set was divided in 45 groups according to their abundance. The average Na4vSS value of each group was calculated and the two parameters were compared (Figure 2D). A significant correlation was observed (R = 0.71), indicating a relationship between the polypeptide solubility and the abundance levels in the cytosol. This correlation suggests an evolutionary selection of bacterial cytoplasmic proteins to minimize their deposition at the concentrations required for their proper biological functions. The higher solubility of high-abundant proteins would work to prevent the aggregation of these proteins even if they become concentrated at specific sub-cytosolic locations. Moreover, because of their high concentrations, their low deposition propensity would contribute significantly to decrease the overall cytosol aggregation tendency and prevent the initiation of spontaneous, non-specific aggregation processes that can deplete the cell of less represented and/or functionally important proteins.

### The Intrinsic Properties of High-Abundant Proteins Decrease Their Aggregation Propensities

**Figure 3:**
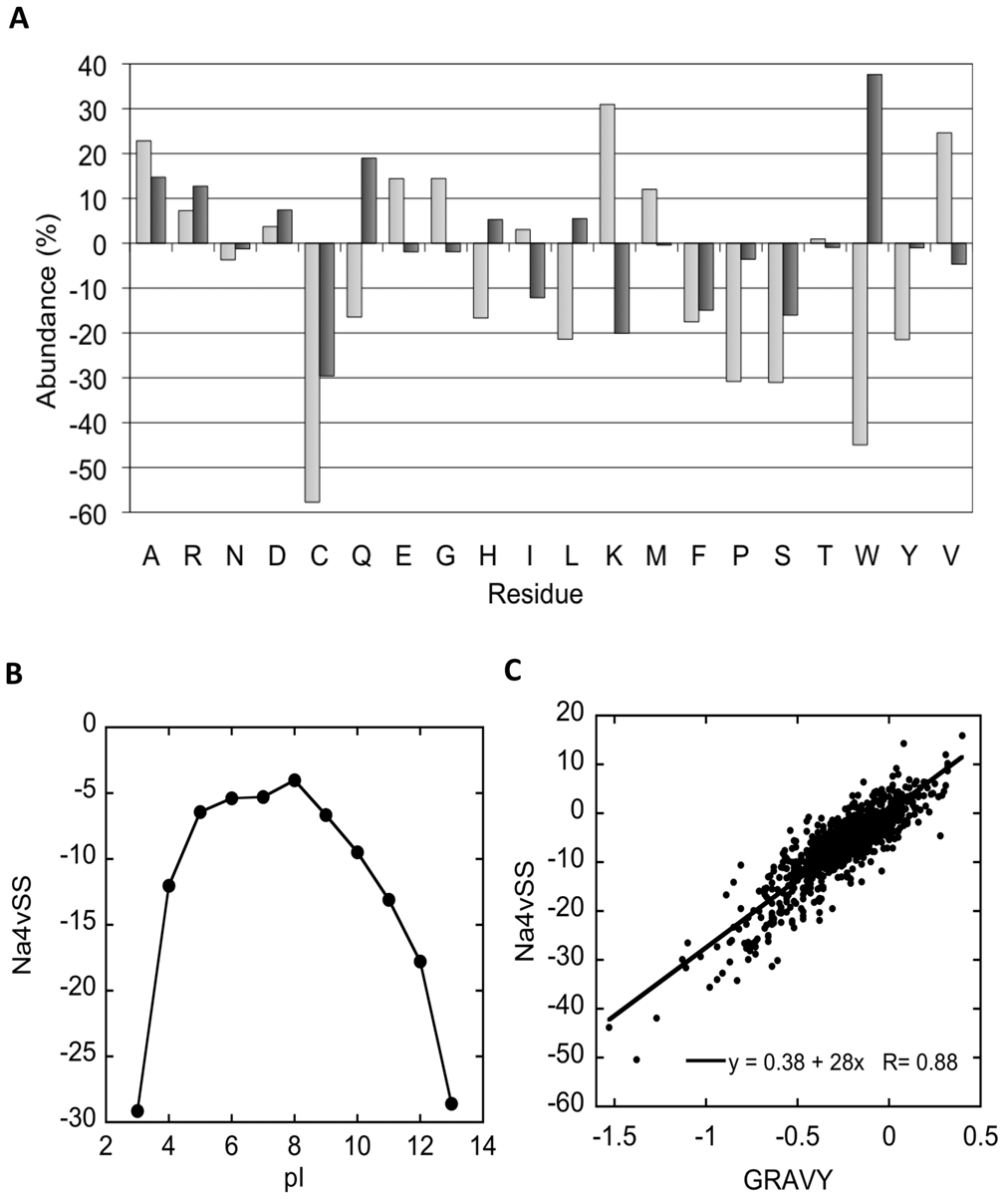
Relationship between the cytosolic proteins abundance and their intrinsic properties. A) Amino acid abundance in high-abundant (pale grey) and low-abundant (dark grey) sequences relative to the expected frequencies in natural proteins as deduced from Swiss-Prot [82]. B) Comparison between the proteins pI and Na4vSS values. C) Correlation between proteins hydropathicity (GRAVY) and Na4vSS values.

The results suggest that the high-abundant proteins would be less aggregation-susceptible than low-abundant ones not because they have fewer or weaker aggregation-prone regions, but because these segments are located in a much more soluble sequence context, which counteracts their self-assembly tendency. Therefore, we analysed whether the two groups of sequences differed in their amino acid composition (Figure 3A). One of the most striking differences between the compositions of the two protein sets is a strong bias for a higher presence of Lys residues in the high-abundant protein set. Also, Glu is more represented in this set, but the difference compared to the low-abundant protein set is lower than in the case of Lys. The other charged residues, Arg and Asp, are found in similar amounts in both protein sets. This causes the overall theoretical isoalectric point (pI) of high-abundant proteins (8.48) to be higher than that of low-abundant ones (6.71). The pH of the *E. coli* cytosol is thought to be around 7.5 [38]. Accordingly, the overall deviation from the physiological pH is higher for the high-abundant protein set (+0.98 units) than for the low-abundant group (−0.79 units). We analysed the individual contributions of polypeptides to these deviations by measuring the percentage of proteins whose pI deviated two pH units below or above the physiological pH. According to this criterion, highly acidic and basic polypeptides constituted 27% and 53% of high-abundant proteins, respectively; in contrast, to 20% and 10% in the low-abundant protein set. This means that, as a general trend, low-abundant proteins have a pI closer to the cytosolic pH than those high-abundant. To test whether there is any relationship between the theoretical pI of a protein and its predicted deposition propensity, we grouped the polypeptides in the cytosolic fraction according to their pIs. Then the average Na4vSS was calculated for each group and plotted against the pI. The resulting graphic shows that proteins with a pI distant from the bacterial cytosolic pH, either more acidic or more basic, have lower aggregation propensities (Figure 3B), explaining why high-abundant proteins tend to populate the extremes of the pI distribution. Because the net charge of a protein at a given pH depends on its pI, these results are in agreement with previous observations indicating that, *in vitro*, the net charge of a protein anti-correlates with its aggregation propensity [39], [40], [41].

The abundance of both acidic and basic proteins in the high-abundant proteins can be attributed to the overrepresentation of Glu and especially Lys residues and suggests that these excesses of charged residues do not mutually compensate for each other in this protein group. Importantly, Lys is by far the least frequently buried residue among the 20 natural amino acids [42]. This is because it needs two other residues to hydrogen bond to its side chain nitrogen atom when it is located in the core of the protein. Glu residues are also less frequently buried in the core than Asp because they have a weaker tendency to bond to the local main chain. This suggests that in high-abundant polypeptides, these residues are preferentially located at the surface in the folded conformation. Interestingly enough, it has been recently shown that increasing the net charge in the surface of a globular protein is a very effective strategy to prevent its aggregation, even in harsh conditions [43], [44]. It is likely that the *E. coli* cytosol would exploit the same strategy to prevent the aggregation of highly abundant polypeptides.

Apart from the charge, another property that strongly influences the overall aggregation propensity of a protein sequence is its hydrophobicity [2], [25], [45]. Interestingly, the proportion of hydrophobic residues in these two groups is not dramatically different: 41.6% and 42.4% for high-abundant and low-abundant proteins, respectively. However, a bias toward the presence of larger residues, like Trp or Tyr, in the place of smaller residues, like Val, is observed in low-abundant proteins (Figure 3A). This suggests that low-abundant polypeptides could be overall more hydrophobic. We used the grand average of the hydropathicity (GRAVY) as measure of the hydrophobicity of both protein sets [46]. The average GRAVY scores are −0.24 and −0.36 for low- and high-abundant proteins, respectively. Also, 38% of high-abundant polypeptides have a GRAVY value below -0.5, in contrast with only 10% of low-abundant ones. Both data indicate that high-abundant proteins tend to be less hydrophobic than low-abundant. This is likely because hydrophobicity is strongly associated with the aggregation propensity, as shown when analyzing the correlation between these two parameters in the complete cytosolic set (R = 0.88) (Figure 3C). It is worth mentioning that Cys residues are underrepresented in both cytosolic protein sets, but especially in the high-abundant set, relative to the conjunct of natural proteins. Reducing conditions prevail in the cytoplasm and disulfide bonds do not normally form correctly in this compartment, which can result in the accumulation of misfolded and inactive proteins [47]. The low content of Cys in bacterial cytosolic proteins is likely the result of a negative selection to avoid these phenomena.

### Gene Expression Levels and Cytosolic Proteins Aggregation Propensities Are Anti-Correlated

**Figure 4:**
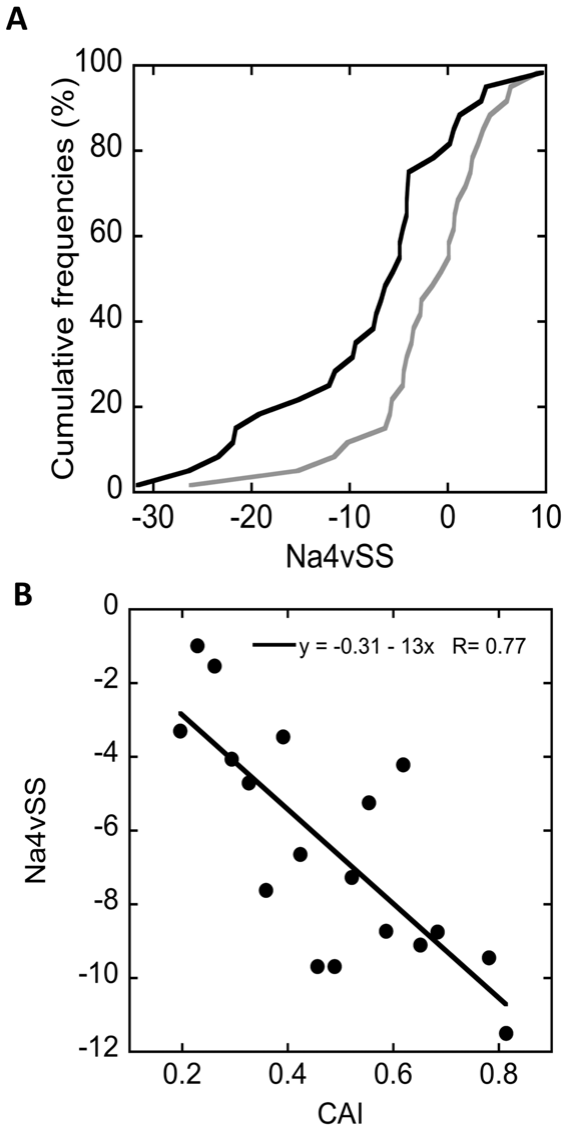
Comparison between cytosolic proteins theoretical expression levels and their aggregation parameters. A) Cumulative distributions of Na4vSS values in the 10% cytosolic proteins with the highest (black) and lowest (grey) Codon Adaptation Index (CAI) values. B) Correlation between the CAI and the Na4vSS values. Each point represents the average value over all the sequences having a CAI value comprised in an interval of 0.03.

The correlation between the effective protein concentration and aggregation propensity suggests that this relationship is controlled at the gene level, providing the cell with the versatility and adaptability necessary to react to different environmental conditions and/or cellular states. However, mRNA and protein abundances do not necessarily exhibit a strong correlation [48]. We compared theoretical expression levels and aggregation propensities to test if the observed correlation at the protein level applies also for gene expression. The codon usage can be employed to approximate the theoretical protein expression levels, obtaining similar estimations to those derived from quantifying mRNA abundance [49], [50]. We used the codon adaptation index as a measure of the codon usage. Low values are associated with low expression levels and high values correspond to high expression levels [29]. The comparison of the 10% of genes encoding cytoplasmic proteins with the higher and lower values shows that both sets present distinctive aggregation features. The low expressed group presents higher Na4vSS values than the highly expressed one (Figure 4A). In addition, when all the cytoplasmic proteins are arranged into 20 groups according to their codon adaptation indexes, a significant correlation between this parameter and the protein aggregation propensity (R = 0.77) is observed (Figure 4B).

These results are in agreement with those obtained using emPAI as a measure of the experimental protein concentration, which overall suggests that the relationship between the protein concentration and aggregation propensity is controlled at the gene expression level. Confirming this hypothesis, a relationship between the mRNA expression levels and protein solubility in *E. coli* has been recently described [51]. Beginning with the AGGRESCAN scale, Tartaglia and co-workers also observed that sequences with the highest mRNA expression levels are less aggregation-prone and *vice versa*. Importantly, this anti-correlation also applies for human proteins [30], [52] suggesting, that, in general, and across the different realms of life, the degree of protein solubility is sharply adjusted to the gene expression levels required for an optimal cell function. This implies that there is little margin of response in front of changes that decrease intrinsic solubility or increase expression levels [52], both effects resulting in an increased aggregation probability.

### Soluble Recombinant Proteins Resemble Cytosolic High-Abundant Proteins

We have previously shown that recombinant soluble proteins have, on average, lower aggregation propensities than those that accumulate as insoluble deposits in the bacterial cytosol upon heterologous overexpression [10]. Extending this observation, Tartaglia and co-workers were able to theoretically forecast the solubility of recombinant proteins in bacteria from their expected expression levels [51]. These data converge to indicate that successfully expressed recombinant proteins would resemble the high-abundant more than the low-abundant proteins. The sum of the squared differences between the amino acid composition of a set of soluble recombinant proteins [10] and that of the high-abundant and low-abundant groups is 79.5 and 114.9, respectively, thus providing support for this hypothesis.

### A Relationship between Protein Molecular Weight and Aggregation Propensity

**Figure 5:**
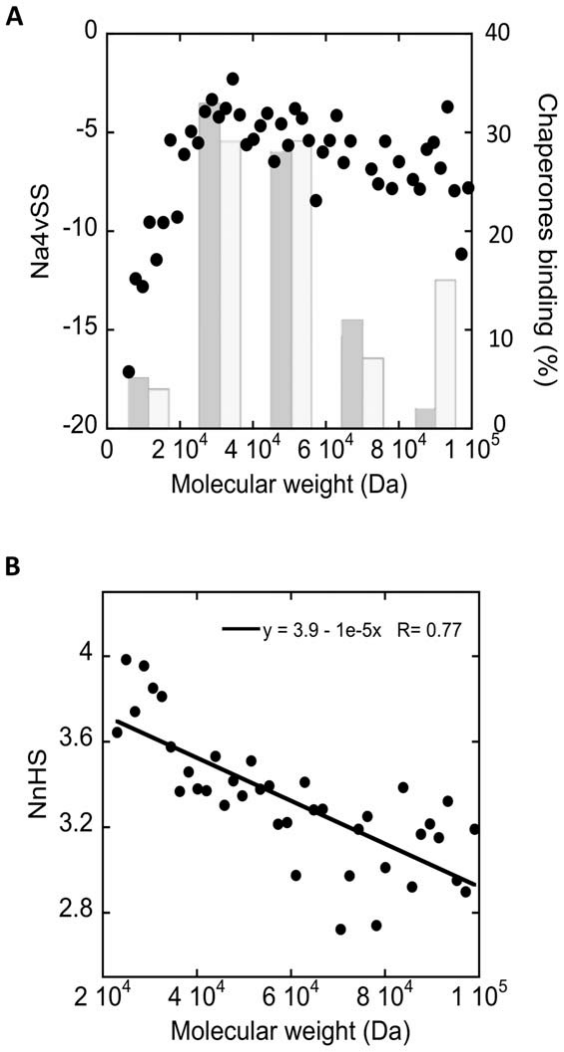
Dependence of proteins length on their aggregation properties and chaperone binding affinity. A) Dot plot distribution represents the relationship between the molecular weight and Na4vSS. Columns show the size distribution of polypeptides that bind to GroEL (grey) or DnaK (white) in *E. coli* according to the data in [61]. B) Relationship between the molecular weight and the NnHS. Each point corresponds to the average value over all the sequences having a length comprised in an interval of 1.9 kDa.

Chiti and co-workers have recently suggested that long human protein sequences have been shaped by evolution in order to reduce their intrinsic aggregation properties [21]. To study the relationship between the protein size and deposition propensity in bacterial cytosolic proteins, we grouped proteins into 50 sets according to their molecular weights (MW) and the average Na4vSS for each particular group was calculated. As shown in Figure 5A, the nature of the relationship between the aggregation propensity and protein length depends on the particular size of the polypeptide. For small proteins, up to approximately 20 kDa in size, the increase in MW is associated with a rapid increase in the aggregation propensity (R = 0.92). Once this size limit is over-passed, the correlation is inverted and further increases in size are linked to a predicted slow, but progressive, increase in solubility (R = 0.75). If we consider the shape of a protein close to a sphere, then its surface area would be approximately proportional to the two-thirds power law of its volume [53]. This implies that, for globular proteins, the relative size of the core grows with protein size [54]. Because hydrophobic residues usually occupy the core of the protein to avoid interaction with water molecules, it is deduced that the proportion of hydrophobic residues, and therefore the overall aggregation propensity, increases with the protein size. Nevertheless, in real proteins, the correlation between the protein size and the fraction of hydrophobic amino acids appears to apply only for proteins until 170 residues [42], in agreement with the observation that the aggregation propensity attains maximum values in this size range. The protein aggregation propensity might act as a determinant of protein size and could be the underlying reason explaining why, above the ∼20 kDa limit, the ratio between hydrophobic and hydrophilic residues does not increase significantly with size [55], [56]. An important implication of the volume/surface relationship in globular proteins, is that, if the proportion of hydrophobic residues is approximately constant, the number of polar residues buried inside the structure should increase with protein size [55], [57], [58]. Because charged residues are more hardly accommodated inside proteins than other polar residues, long proteins tend to have fewer charges [59], which together with their slow folding rates [60], would make these proteins aggregation susceptible. According to our data, in *E. coli* polypeptides, these effects are partially compensated by an overall decreased sequence aggregation propensity. Importantly, above the 20-kDa limit, the NnHS values steadily decrease with the protein size indicating that in longer proteins (Figure 5B), the aggregating regions tend to be more distant in the sequence. Interestingly enough, the main bacterial chaperones, GroEL and DnaK, interact poorly with proteins smaller than 20 kDa and display a preference for larger substrates (Figure 5A) [61], [62], [63], suggesting the presence of redundant mechanisms to reduce the aggregation propensity of long bacterial proteins, as previously described for the human proteome [21].

### The Composition of Hot Spot and Gatekeeper Stretches

**Figure 6:**
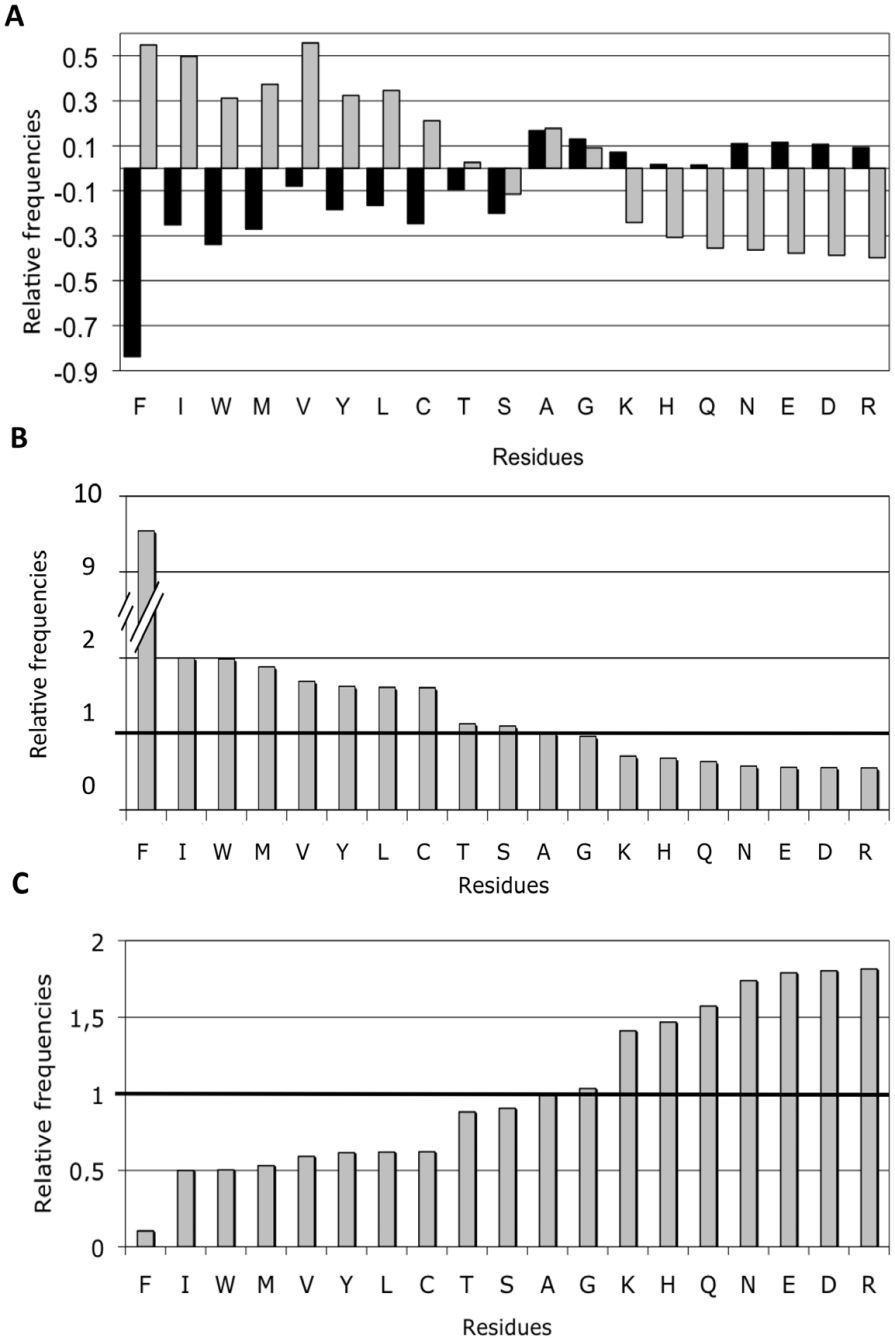
Amino acid composition of cytosolic proteins hot spots and their flanks. A) Amino acid frequencies relative to their average frequency in natural proteins as deduced from Swiss-Prot [82]. A relative frequency of 0 for a given residue at a given position means that the residue occupies that position with a frequency identical to that in natural proteins. Residues enrichment in the hot spots (B) and at the flanks (C) relative to their frequency in natural proteins. Values above or below 1.0 point denote increases or decreases in frequency, respectively.

It has been suggested that evolution exploits negative design principles to modulate protein deposition by placing residues that counteract aggregation at the flanks of hot spots [21], [27], [64]. These residues would act as gatekeepers [27] and reduce the protein propensity to self-assemble into macromolecular aggregates. At the same time, it appears that the cellular quality control has evolved to recognize and block these sequence patterns [21], [27]. Accordingly, several disease-associated mutations have been linked to the disruption of gatekeeper stretches [65]. To confirm these observations, we proceeded to study whether, in bacterial cytosolic proteins, aggregation-prone segments and their flanking sequence stretches differ in composition (Figure 6A). The comparison of the amino acid frequency in the these regions with their natural abundance shows that hydrophobic and aggregation-promoting residues (Val, Phe, Ile, Tyr Met and Leu) are overrepresented inside HS and, on the contrary, that flanking regions are enriched with polar and soluble residues (Arg, Asp, Glu, Asn Lys and Gln). The rate between the frequency of each amino acid inside aggregation-prone sequences and at the flanks evidenced that Phe displays a high preference for being a component of aggregation-prone regions (Figure 6B). In contrast, the charged Arg, Lys, Asp and Glu residues display a high preference for being at the flanks (Figure 6C). The gatekeeper action of these residues is exerted through the repulsive effect of the charge (Arg, Lys, Asp and Glu) and the increase in entropy penalties upon assembly (Arg and Lys). Our data are in agreement with the distribution found using the TANGO and Zygregator algorithms on the theoretical *E. coli* and human proteomes [21], [27], indicating that the protective action of the flanking residues acts on the combination of proteins that are being effectively expressed in the bacterial cytosol. As described above, another important gatekeeper residue is Pro, which acts as a beta-breaker. Because AGGRESCAN considers the presence of a Pro residue in a sequence stretch incompatible with this sequence being a hot spot, its frequency could not calculated.

### The Relationship between the Aggregation Propensity and Protein Function in Cytosolic Proteins

**Figure 7:**
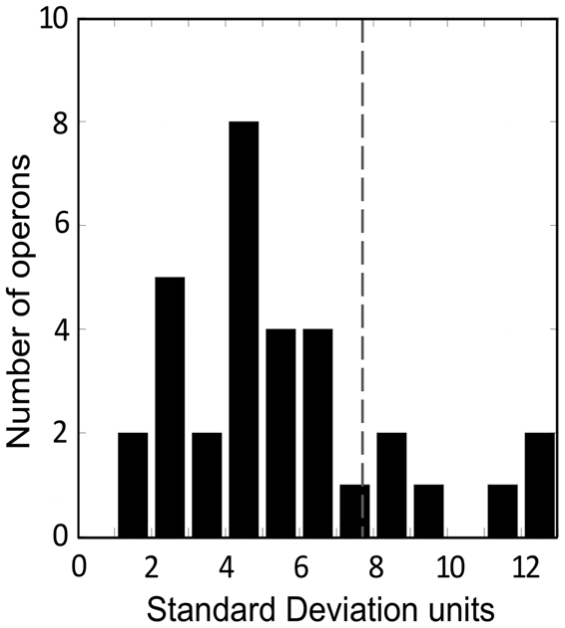
Proteins encoded by the same operon display related aggregation propensities. Standard deviation of Na4vSS values in the 25 analysed operons. The standard deviation in the complete cytosolic set is 7.72 (dashed line). Low standard deviation within an operon indicates that the aggregation propensity of its proteins is similar.

The set of genes in an operon share a common gene expression regulation and are generally connected by their biological function. As a result, proteins encoded by the same operon are suggested to be present in similar amounts in the cell [29]. The observed association between protein aggregation and abundance would imply that polypeptides in the same operon should have related aggregation propensities. In agreement with this hypothesis, the standard deviation of the Na4vSS value between proteins regulated by the same operon is lower in 78% of the cases (25 of 33) than the standard deviation in the complete set of proteins (7,72 Na4vSS) that could be ascribed to a particular operon (Figure 7). This suggests again a link between protein aggregation propensities and the rates of transcriptional initiation.

The impact of protein aggregation on cellular function would be ultimately associated to individual fitness. Therefore, it is conceivable that evolution would select for an overall decreased aggregation propensity in operons performing essential cellular functions. To explore this possibility, the bacterial operons where divided in two groups according to their Na4vSS values, those with lower and higher aggregation propensity than the mean propensity of the complete operon protein set (−6.4 Na4vSS). The essentiality of approximately half of the proteins in each subset has been annotated via genetic footprinting or knockout experiments [66], [67]. Importantly, considering only the annotated polypeptides, operons with low aggregation tendency regulate 85% of essential proteins and 15% of nonessential ones. In contrast, operons with high aggregation propensity encode a similar proportion of essential and nonessential proteins, 48% and 52% respectively (Table 1), suggesting that the sequences of essential bacterial cytoplasmic proteins suffer a stronger selection against deposition than those of nonessential ones, as previously proposed for different eukaryotic organisms [68].

**Table 1:** Different operons regulate proteins with different aggregation propensity and biological function.

**LA operons name** a	**Na4vSS**	**n° proteins**	**Ribosomal**	**Essential**	**Non-essential**	**Unknown**
**yjeFE-amiB-mutL-miaA-hfq-hflXKC**	−15.63	3	0	1	2	0
**hscBA-fdx**	−14.33	3	0	2	0	1
**rpsMKD-rpoA-rplQ**	−14.32	5	4	3	0	2
**cmk-rpsA-himD**	−13.20	3	1	0	0	2
**rpsF-priB-rpsR-rplI**	−12.93	3	3	2	0	1
**pheST-himA**	−12.50	3	0	0	0	3
**rpsLG-fusA-tufA**	−11.70	3	2	2	0	1
**rpsJ-rplCDWB-rpsS-rplV-rpsC-rplP-rpmC-rpsQ**	−11.47	11	11	4	0	7
**thrS-infC-rpmI-rplT**	−11.25	4	2	0	0	4
**metY-yhbC-nusA-infB-rbfA-truB-rpsO-pnp**	−11.17	7	1	4	0	3
**iscRSUA**	−9.78	4	0	2	1	1
**rpsP-rimM-trmD-rplS**	−8.60	4	2	3	0	1
**rplNXE-rpsNH-rplFR-rpsE-rpmD-rplO-prlA-rpmJ**	−8.52	9	9	3	0	6
**aroKB-damX-dam-rpe-gph-trpS**	−7.60	3	0	2	0	1
**galETKM**	−7.47	3	0	0	2	1
	**Total**	**68**	**35**	**28**	**5**	**34**
		**%**	**51.47**	**41.18**	**7.35**	**50.00**
**HA operons name** b	**Na4vSS**	**n° proteins**	**Ribosomal**	**Essential**	**Non-essential**	**Unknown**
**ribF-ileS-lspA-slpA-lytB**	−5.97	3	0	1	1	1
**rplJL-rpoBC**	−5.93	4	2	0	0	4
**nuoABCEFGHIJKLMN**	−5.87	3	0	0	1	2
**sdhCDAB-b0725-sucABCD**	−5.74	5	0	2	2	1
**leuLABCD**	−5.55	4	0	0	0	4
**entCEBA-ybdB**	−5.54	5	0	0	4	1
**minced**	−4.50	3	0	2	0	1
**fabHDG-acpP-fabF**	−4.38	4	0	4	0	0
**gcvTHP**	−4.13	3	0	0	0	3
**dhaKLM**	−4.03	3	0	1	0	2
**ptsHI-crr**	−3.33	3	0	0	1	2
**deoCABD**	−3.23	4	0	0	1	3
**thiCEFGH**	−2.53	4	0	0	1	3
**hisGDCBHAFI**	−1.87	3	0	0	0	3
**mraZW-ftsLI-murEF-mraY-murD-ftsW-murGC-ddlB-ftsQAZ**	−1.68	4	0	3	0	1
**rfbBDACX**	−0.86	5	0	0	3	2
**gatYZABCDR_2**	5.90	4	0	1	1	2
	**Total**	**64**	**2**	**14**	**15**	**35**
		**%**	**3.13**	**21.88**	**23.44**	**54.69**

*a Operons regulating proteins with aggregation propensity lower (LA) than the mean aggregation propensity of the complete operon protein set (−6.4 Na4vSS).*

*b Operons regulating proteins with aggregation propensity higher (HA) than the mean aggregation propensity of the complete operon protein set (−6.4 Na4vSS).*

**Figure 8:**
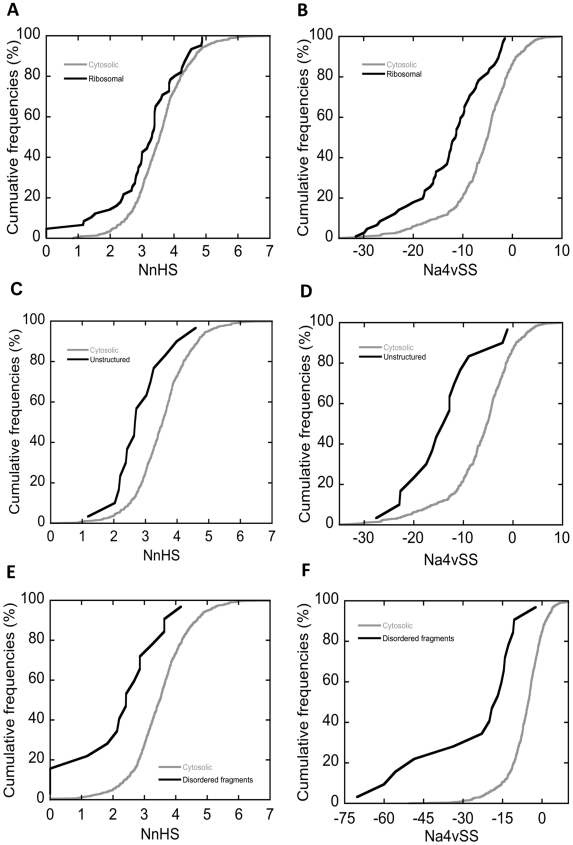
Disordered sequence stretches display reduced protein aggregation. Cumulative distributions of NnHS and Na4vSS values in ribosomal proteins (A and B), intrinsically unstructured proteins (C and D) and disordered fragments in cytosolic proteins (E and F) are compared with the distribution in the complete cytosolic set (grey).

A deeper analysis of the two operon subsets reveals that operons with associated low aggregation propensity control the expression of 95% of the bacterial ribosomal proteins that could be ascribed to a given operon (Table 1). This suggests, because of their crucial function, ribosomal proteins might display differential aggregation traits. The analysis of the 53 ribosomal proteins detected in the cytosolic extract shows that these polypeptides display fewer aggregating segments and lower Na4vSS values than the rest of proteins in the bacterial cytoplasm (Figures 8A and 8B). Low aggregation propensities have been also predicted for human ribosomal proteins [21]. Tartaglia and Vendruscolo have recently shown that human proteins in small cellular localisations tend to have low aggregation propensities, being the polypeptides residing at the ribosome the ones confined in the smallest volume and having the highest associated average solubility [30]. The same principle seems to apply for the bacterial ribosome proteins, suggesting a common evolutionary pressure for highly soluble ribosomal proteins.

Ribosomal proteins are commonly characterised by the presence of unstructured sequence stretches. These regions act as “structural mortar”. They have evolved to bind the ribosomal RNA and thereafter acquire a partial ordered structure that fills the gaps of the ribosome structure [69]. These unstructured regions might confer ribosomal proteins with a lower aggregation propensity than the rest of the cytosolic domains, in line with the idea that disordered sequences have been evolutionary selected to avoid the presence of aggregation-prone residues as a strategy to prevent the self-assembly of the fully solvent-exposed polypeptide chain in the absence of a protective secondary structure [22]. To confirm that this relationship applies for bacterial cytosolic proteins, we identified those polypeptides classified as intrinsically unstructured (IUP) according to the Disprot Database [70], calculated their aggregation parameters and compared them with the rest of cytosolic proteins (Figures 8C and 8D). As expected, bacterial cytosolic IUPs present a significantly decreased aggregation propensity. The difference in the aggregation propensity between the folded and disordered protein regions becomes even clearer if we only consider the fully unstructured sequences in IUPs and not the whole protein (Figures 8E and 8F). Very similar results were obtained when we analyzed the 32 proteins in the cytosolic fraction predicted by the FoldUnfold algorithm [19] to be intrinsically unstructured (data not shown).

Computational analysis suggest that, on the average, proteins in the bacterial cytosol are more aggregation prone than those in the human cytosol [30], which is in agreement with the hypothesis that organisms with simpler cellular organisation and shorter life span have, as a trend, higher aggregation propensities [24]. Because, IUPs tend to be more soluble than their globular counterparts, independently of the analyzed proteome, the higher proportion of unstructured proteins in the proteomes of higher organisms, and specifically in humans, might well account for the lower aggregation propensities of their cytosolic protein ensemble.

### Bacterial Proteins in the Periplasm and Inner and Outer Membranes Possess Characteristic Aggregation Propensities

Eukaryotic cells consist of a complex collection of compartments characterised by different environmental conditions and molecular compositions [71], [72]. It is suggested that proteins located in a particular eukaryotic subcellular location have been evolutionary selected to fold and avoid protein aggregation in this environment [21], [22], [23], [24]. Bacterial proteins are found in other compartments apart from the cytosol, like the periplasm and the inner and outer membranes. Presumably their aggregation properties would be also adapted for their optimal function at those subcellular locations. As described above, the original data set used in the present work was enriched in cytoplasmic proteins but contained also polypeptides assigned to other cellular places. We took advantage of this protein diversity to analyse the aggregation properties of proteins residing in different compartments.

**Figure 9:**
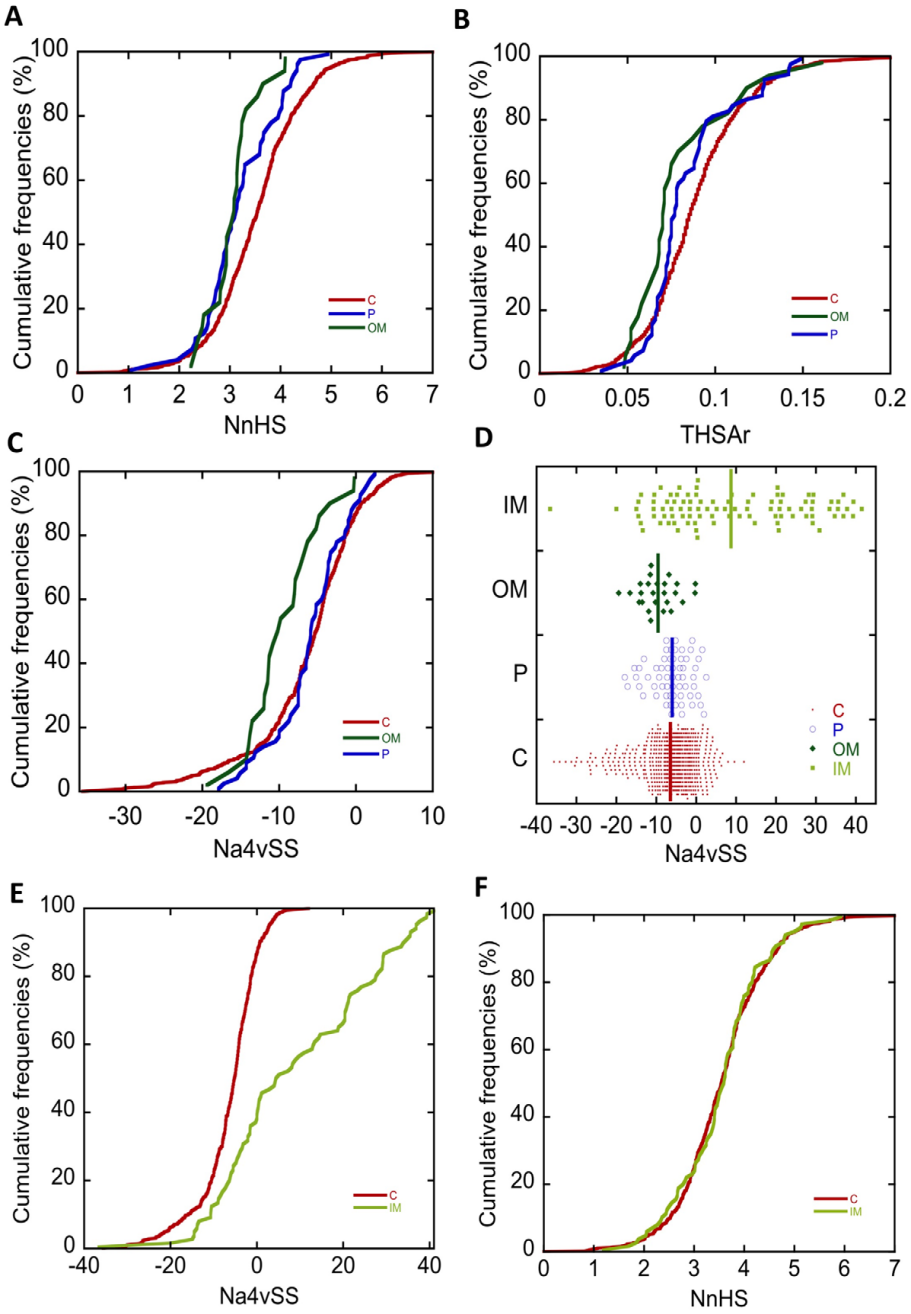
Relationship between subcellular localisation and protein aggregation propensity. Cumulative distribution of NnHS (A), THSAr (B) and Na4vSS (C) of proteins located in the cytoplasm (C, red), outer membrane (OM, dark green), periplasm (P, blue). D) Dot distribution of the Na4vSS values of the proteins in the previous four protein sets as well as those located in the inner membrane (IM, pale green); the vertical lines correspond to the Na4vSS mean in each protein set. Cumulative distribution of NnHS (E) and Na4vSS (F) in cytosolic and inner membrane proteins.

Cytoplasmic and periplasmic proteins exhibit a similar average aggregation propensity although a sharper distribution of Na4vSS values was observed in the periplasm, in which proteins with extreme aggregation propensities were absent (Figure 9). The number of aggregation-prone fragments and their associated areas are lower in periplasmic proteins, suggesting that despite having a content of aggregation-prone residues similar to that of cytosolic proteins, these residues are differently arranged in the sequence (Figure 9). This is consistent with the observation that the average number of alternating hydrophobic/hydrophilic stretches (>5 residues) is 30% higher in periplasmic proteins, which might indicate a tendency to reduce the presence and impact of contiguous aggregation-prone regions. In line with this hypothesis, Chang and co-workers demonstrated experimentally that periplasmic proteins are preferentially resistant against aggregation under denaturing conditions and that this behaviour is not related to a higher thermodynamic stability, but rather to sequence characteristics [73]. This property can be evolutionary advantageous in the periplasm that, in contrast to the cytosol, lacks a sophisticated cellular system to control protein quality and avoid aggregation [72] and is separated from the outside solution by a highly permeable outer membrane that provides limited protection against environmental variations. In addition, taking into account that the volume of the periplasm (0,065 µm^3^) is ten fold smaller than that of the cytoplasm (0,67 µm^3^), the results suggest that the inverse correlation observed in human tissues between the size of the cellular compartment and the aggregation propensity of the proteins that reside in it [30], also applies in the less compartmented bacterial background.

**Figure 10:**
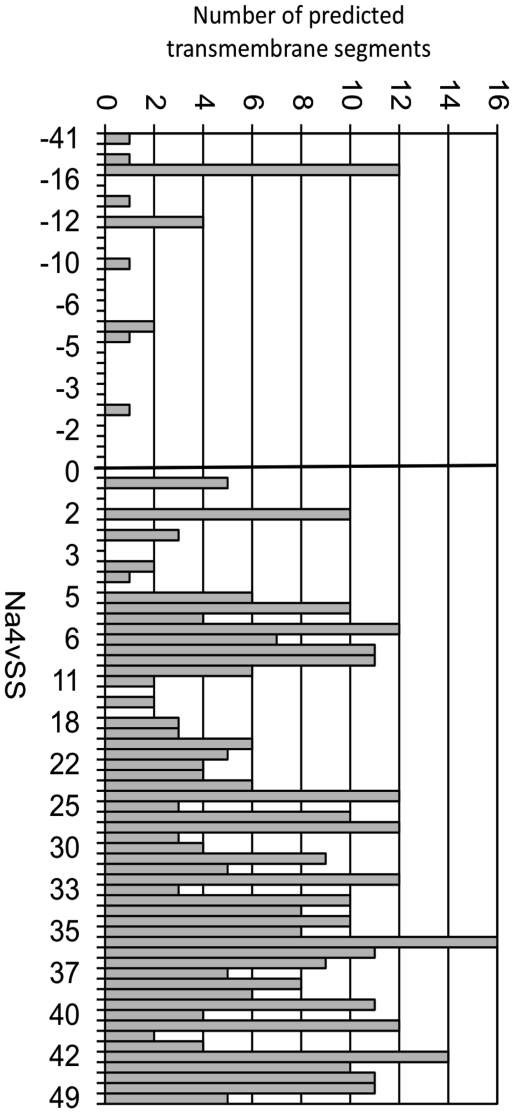
The inner membrane contains proteins with different number of transmembrane segments and associated aggregation propensities. Diagram of the inner membrane protein set showing the Na4vSS value and the number of transmembrane segments.

**Figure 11:**
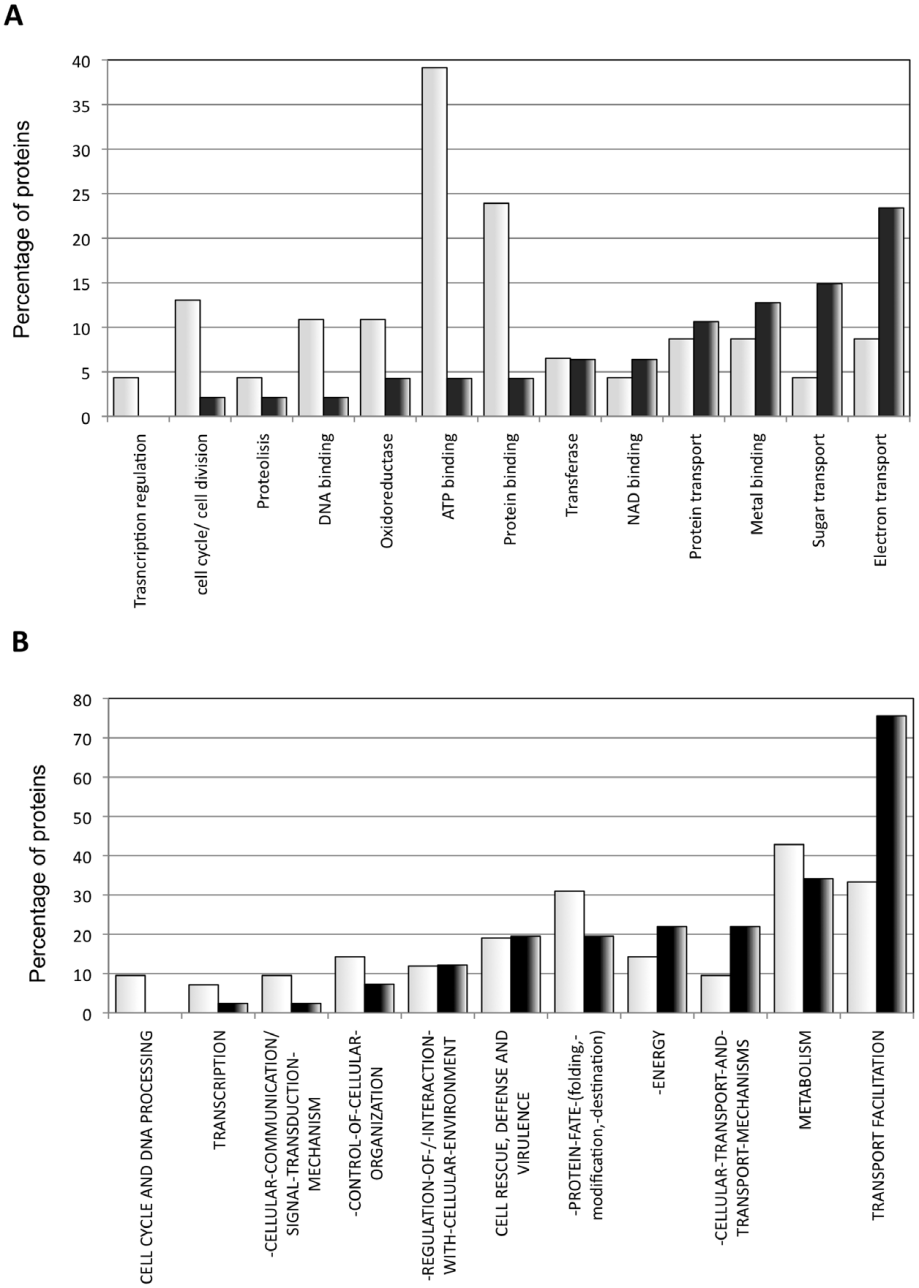
Inner membrane proteins with differential aggregation propensities are involved in different biological functions. Percentage of inner membrane proteins associated with the biological functions described in FunCat (A) and UniProtKB (B). The inner membrane proteins were divided in two groups according to their Na4vSS value: Na4vSS <6 (42 proteins; pale grey) or Na4vSS ≥6 (43 proteins; dark grey).

The gram-negative bacterial inner membrane is a semipermeable shield that preserves the cytoplasm environment. The proteins associated with the this membrane are principally composed of α-helices and could have a variable number of transmembrane segments (TS) per protein [71]. These regions are stable in the hydrophobic environment of this lipid bilayer due to a primary sequence rich in apolar residues. In this sense, it is necessary for a protein to have a stretch of 15–25 residues to transverse the membrane bilayer. Consequently, the extraction and analysis of these proteins in aqueous solvents frequently causes aggregation problems [71]. In agreement with these data, AGGRESCAN shows that inner membrane proteins possess the highest aggregation propensities of all bacterial proteins (Figure 9C). Surprisingly, inner membrane proteins contain a number of hot spots similar to that in cytoplasmic proteins (Figure 9D). However, in the inner membrane proteins, the area associated to these hot spots is much larger, indicating that they are significantly longer and/or contain more aggregation-prone residues (Figure 9E). These results are consistent with the observations obtained with TANGO, which also showed that membrane-associated proteins do not contain a higher amount of beta-aggregation nucleating regions than the proteins located in the cytoplasm [22]. Interestingly, when the Na4vSS values of inner membrane proteins were plotted as a dotted distribution, the existence of two protein groups become evident: a first group with an aggregation propensity similar to that of cytosolic proteins and a second group with particularly high Na4vSS values (Figure 10). We found that the main difference between these groups is the number of TS. The TMHMM version 2.0 [74] program calculated that 83% of the proteins in the first group contain fewer than three TS whereas 89% of the second group has more than three TS (Figure 9, Table 1). To decipher whether the different aggregation propensities exhibited by these two protein subsets was associated with particular biological functions (Figure 11), we consulted the functional descriptions collected in the Functional Catalogue Database (FunCatDB) [75] and in the Protein Knowledgebase (UniProtKB) [76], [77]. According to the FunCatDB, proteins with high Na4vSS are preferably related to “transport facilitation” whereas functions like “cellular communication” or “protein fate” appear to be associated with membrane proteins displaying lower aggregation propensities. In agreement with these data, according to UniProtKB, membrane proteins with a high aggregation propensity are preferentially involved in “electron transport” and “sugar transport” whereas proteins with low Na4vSS are associated to processes like “protein binding” and “ATP binding”. Because, according to our analysis, inner membrane proteins with high aggregation propensities also contain many TS, they must be totally inserted in the membrane, limiting their actions to functions principally related to transport and respiratory activities. In contrast, polypeptides with low aggregation propensities are anchored in the membrane by only one or two transmembrane helices, the rest of the protein being available to assume different biological activities like signal transduction [78].

Outer membrane proteins are thought to be located in a hydrophobic environment, and consequently, they are expected to have a high aggregation tendency. However, they exhibit a low aggregation propensity according to all AGGRESCAN parameters (Figure 9). In fact, the outer membrane acts as a permeable barrier to hydrophobic substances. In general, outer membrane proteins display a beta barrel structure that encloses a hydrophilic cavity covered by a hydrophobic outer layer. The presence of an apolar hollow space is essential for their function as porins. Interestingly, this particular assembly is achieved by alternating hydrophobic and hydrophilic segments [79], [80]. As a result, outer membrane proteins display two times more alternating hydrophobic/hydrophilic stretches (>5 residues) than cytoplasmic proteins. The presence of these characteristic polar regions reduces the protein hydropathy and overall aggregation propensity but also limits the number and area associated to aggregation-prone sequence stretches. These properties could be important not only for their biological function but also for their biogenesis. As recently reviewed by Knowles and co-workers, the folding of proteins into the outer membrane presents important challenges to Gram-negative bacteria because they must migrate from the cytosol, through the inner membrane and into the periplasm before they could be recognized by the beta-barrel assembly machinery and inserted into the outer membrane [81]. In most of these steps and compartments, the protein is unfolded and accordingly sequences with reduced aggregation propensities would represent a selective advantage.

In the present study, we have characterized the aggregation properties of an experimentally determined bacterial proteome. The data are consistent with previous observations obtained through the analysis of theoretical proteomes using different computational strategies. In particular, we could confirm that the observed anti-correlation between mRNA levels and aggregations propensities [30], [51], [52] is effectively translated to the protein level in physiologically relevant environments. The data argue that selective pressure against protein aggregation plays an important role in shaping the protein sequence space. In this way, abundant proteins have evolved specific sequence features aimed to increase their solubility in the crowded bacterial cytosol. We could confirm that nature uses negative design principles to avoid the self-assembly of aggregation-prone regions in globular cytosolic proteins as well as the strongly decreased aggregation propensity of cytosolic IUPs, as previously proposed by Serrano and Chiti groups by analyzing different virtual proteomes [21], [22], [27]. Our data demonstrate that, as in humans [21], the evolution of long bacterial protein sequences has been constrained to reduce their aggregation propensity, suggesting a general rule that applies independently of the organism complexity. Importantly, this feature appears to have coevolved coordinately with the size recognition preferences of the chaperone complement present in each particular organism [21]. The analysis of the operons aggregation propensity shows that, as previously shown in eukaryotes [23], [68], bacterial proteins executing important cellular functions tend to be better adapted against aggregation than nonessential ones, suggesting again a generic mechanism to improve cellular fitness in normal physiological conditions but specially in front of stress. Finally, we could confirm that, as in humans [21], [30] and yeast [23], in bacteria, proteins residing in different compartments display specific aggregation features, suggesting a preferential adaptation to each particular subcellular environment, that as proposed by Tartaglia and Vendruscolo might well be related to the volume of the considered compartment [30].

Overall, our results confirm the general validity of bioinformatic analyses to elucidate the mechanisms by which evolution tunes protein aggregation properties. Together, the results of such analyses argue that aggregation propensity acts as strong constraint during evolution, shaping different polypeptide properties. Accordingly, redundant natural mechanisms to avoid protein aggregation in biological contexts appear to exist. In turn, it is likely that the analysis of the aggregation properties of natural bacterial proteins would provide useful lessons to rationally manipulate and control the production of recombinant proteins in the bacterial cytosol.

## Materials and Methods

### Databases and Parameters Calculation

The amino acid sequences of bacterial proteins were obtained from Swiss-Prot Protein knowledgebase [82]. The protein subcellular location was obtained from PSORT database, version 2.0 [34], [35].

The functions associated with the different sequences in the study were identified using the hierarchically structured functional catalogue (FunCat) [75] and the Protein Knowledgebase (UniProtKB) [76], [77]. FunCat provides a set of functional categories, from 25 catalogued, for each classified protein. The biological processes associated with the different protein sets were assigned according to the ontology information in the TrEMBL database at the UniProtKB server. The essentiality of the bacterial proteins for the cellular fitness was derived from the data reported in [66], [67].

The Database of Protein Disorder (DisProt) (release 4.9) has been used to identify disordered proteins or proteins containing extensive unstructured sequence stretches [70]. DisProt contains 47 *E. coli* proteins experimentally shown to be intrinsically disordered; 20 of them are included in the analysed protein set.

The RegulonDB data base has been used to obtain the known *E. coli* operon structure set [83]. We only considered those operons encoding for at least 3 of the cytosolic proteins in the set.

The average hydropathy score (GRAVY) was calculated using the hydrophobicity values obtained from the Kyte-Doolittle scale [46]. The GRAVY was described as (**∑^n^_i = 1_H_i_**)**/n** where **Hi** is the protein residue hydrophobicity at position **i** and **n** is the protein length.

The number of transmembrane regions was calculated employing TMHMM version 2.0 [74].

The Exponentially Modified Protein Abundance Index (emPAI) of each protein was obtained from the data reported in [29]. The cumulative distribution of the Na4vSS, NnHS and THSAr values associated with the 87 cytosolic polypeptides displaying the highest and lowest emPAI were plotted to analyse their aggregational properties. To analyse the overall correlation between cytosolic proteins abundance and their aggregation propensity we used the logarithm of emPAI LN(emPAI), because, as Na4vSS, it displays in a lineal distribution. The LN(emPAI) comprise values between −2.5 and 23; however there were only 4 proteins between 14 and 23 values and they were discarded for further analysis. The remaining 871 proteins were divided in 45 grups at intervals of LN(emPAI) of 0.37 and the average value of each group calculated. In this way, the different length intervals have similar weights in the correlation, independently of the number of polypeptides present in each group.

The Codon Adaptation Index values were obtained from [29]. The cytosolic polypeptides possess values between 0.19 and 0.83. They were distributed in 20 intervals according to their indexes. Two of these intervals do not contain any protein or only one polypeptide and were discarded to avoid the dispersion of the data distribution. Subsequently the Na4vSS and codon adaptation index average of the 18 remainder groups were calculated.

The pI of the different polypeptides were calculated using the ProtParam tool of the ExPASy proteomics server of the Swiss Institute of Bioinformatics [82].

### Composition of Hot Spots and Flanquing Stretches

Flanquing regions were defined as the 5 residues at the N- and C-sides of a given HS. The frequency of each natural amino acid inside the hot spots and at their flanks was compared with their average frequency in natural proteins as deduced from Swiss-Prot [82]. The relative frequency of a given amino acid in hot spots (F_rh_) was calculated as: **F_rh_ = (F_h_/F_n_)−1** where **F_f_** is its frequency inside the hot spots and **F_n_** its frequency in nature accordingly to Swiss-Prot data base [82]. Values above 1 or below 1 indicate higher or lower frequency, respectively. The same procedure was used to calculate the relative frequency of a given amino acid at the flanks.
